# Physical Activity-Related Injury and Its Associated Factors among Middle School Students in Southern China

**DOI:** 10.3390/ijerph15061244

**Published:** 2018-06-12

**Authors:** Weicong Cai, Yang Gao, Wenda Yang, Fuyuan Cheng, Dongchun Tang, Liping Li

**Affiliations:** 1 Injury Preventive Research Center, Shantou University Medical College, Shantou 515041, China; 16wccai@stu.edu.cn (W.C.); 15wdyang@stu.edu.cn (W.Y.); 13fycheng@stu.edu.cn (F.C.); 17dctang@stu.edu.cn (D.T.); 2 Department of Physical Education, Faculty of Social Science, Hong Kong Baptist University, Hong Kong, China; gaoyang@hkbu.edu.hk

**Keywords:** epidemiology, sports injury, physical activity, risk factor, middle school

## Abstract

Physical activity (PA) promotion is beneficial to gain and maintain optimal health, but might increase risks for physical activity-related injury (PARI). This cross-sectional study aimed to investigate the incidence rate and identify risk factors of PARI among Chinese middle school students. Selected via the method of cluster random sampling, students graded 7–8 (junior) and 10–11 (senior) from five middle schools (aged from 10 to 18 years old) in Shantou were invited to participate in the survey. Information on socio-demography, PA involvement, sleep duration, individual safety awareness and exercise behavior, and PARI experiences in the past 12 month was collected. Multivariate logistic regression model was performed to estimate the risk factors of PARI. A total of 3082 participants completed the valid questionnaires, with an overall incidence rate of 25.1%. Boys, junior school students, sports team members, and those with lower safety awareness, living with single parent, and without any chronic conditions were at higher risks for PARI. Moreover, exercising on wet floor or with illness frequently would also be more likely to experience injury, especially those with at least 120 min per day. In conclusion, PARI was prevalent among middle school students in southern China. The above data provide insights that were focused and effective actions should be taken to prevent school-aged adolescents from PARI and maximize the benefits of PA.

## 1. Introduction

The health benefits of regular physical activity (PA) participation, including muscular fitness, bone health, as well as decreased morbidity and mortality, have been firmly established [1,2,3,4]. Knowledge of these advantages of PA has led almost all countries and regions, including China, to involve in the current global campaign to promote PA [5,6,7]. Meanwhile, the World Health Organization (WHO) has recommended that children and youths aged 5–17 should accumulate at least 60 min of moderate- to vigorous-intensity PA (MVPA) per day with the aim of achieving and maintaining individual health at an optimal level [8].

With the contemporary promotion on physically active lifestyle, however, a corresponding increment of physical activity-related injury (PARI) can be expected [9]. Actually, PARI contributes primarily to non-fatal injury, which ranks as the top one health threat to school-aged adolescents in many countries [10,11,12]. Coupled with other findings, sports and recreational activities were reported to be the cause of as much as 39% of fractures in children and adolescents [13,14]. In addition to the significant financial costs, these injuries could also cause other indirect burden, such as future restriction of PA [15,16,17]. Previous study presented that up to an annual 8% of adolescents discontinued recreational sports owing to PARI events [18]. Additionally, parental concerns could also discourage or prevent their relatively active children from PA involvement [19]. More importantly, adolescents might lose their enthusiasm for involving in healthy PA because of the negative perception and fear of injury risk [20,21]. Those consequences stated above, undoubtedly, are against the initial purpose of PA promotion for public health.

In China, most researchers have reported that sports injuries were not uncommon among school-aged adolescents underlying the fact that the government has been making great efforts to strengthen PA in the youth population in the past decades [5,22,23]. According to earlier studies, age, BMI, PA level and environment, as well as family context were the risk factors of sports injury [9,12,17,18,23]. In addition, there were marked gender and year level differences in the occurrences and severity of injury [12,18]. Nevertheless, studies investigating the epidemiological characteristics of PARI are scarce and the evidence between potential risk factors and injury events from middle school students is limited. Once determined, their important relationships can be applied by both researchers and sport practitioners to implement injury-intervention strategies in targeting vulnerable populations to reduce PARI occurrence. We therefore carried out a cross-sectional study among middle school students in southern China to obtain better understanding about the problem of PARI and further identify significant factors associated with injury experiences that could be of great help for us to take prevention and intervention measures.

## 2. Methods 

### 2.1. Study Sample 

Via the method of cluster random sampling, we selected the schools by the locations and administrative areas of Shantou. Students graded 7–8 (i.e., junior school, aged from 10 to 15 years old) and 10–11 (i.e., senior school, aged from 14 to 18 years old) from five middle schools in Shantou were invited to participate in the study during September and October 2015. The exclusion criteria were as follows: (1) inability to engage in PA; and (2) inability to provide parental consent for participation in the study. 

### 2.2. Data Collection 

Structured questionnaires were distributed to all eligible students in classes by trained personnel. The questionnaire was self-administered and self-reported, consisting of basic characteristics, habitual level of PA, sleep duration, individual safety awareness and exercise behavior, and PARI experiences in the past 12 months. 

The basic characteristics included gender, age, study year, height (centimeter), weight (kilogram), sports team member (yes or no), any diagnosed chronic disease/symptom or not (such as heart disease, vision or hearing disorder), and their parental education levels and marital status.

Participants’ habit of engagement in PA during both inside and outside school per typical week in the past 12 months were asked using the revised CLASS-C (Children’s Leisure Activities Study Survey), which possesses sound reliability (Cronbach’s α = 0.704) in our survey and has been demonstrated to have good internal consistency and 1-week test–retest reliability in the previous study [24]. It was comprised of different kinds of PAs (including playing basketball, hiking, walking, cycling, dancing, etc.) as well as their frequency (total cumulative times on both a typical weekday and a typical weekend, respectively) and duration (total cumulative minutes on both a typical weekday and a typical weekend, respectively). PA volume (cumulative minutes/week) was calculated by the total cumulative minutes on a typical weekday plus that on a typical weekend, and then the minutes of participation in MVPA per day was calculated via PA volume divided by seven. Students were further classified into three categories (i.e., <60 min, 60 to <120 min, and ≥120 min per day, respectively) based on their average daily time of involvement in MVPA.

In terms of sleep time (including nap time), the following two questions were asked: (a) “On average, how many hours do you sleep on a typical weekday?”; (b) “On average, how many hours do you sleep on a typical weekend?”. The average daily time was then generated (using the formula of sum of duration on a typical weekday multiplied by five and duration on a typical weekend multiplied by two, then divided by seven). Participants were further grouped into five categories (i.e., <6 h, 6 to <7 h, 7 to <8 h, 8 to <9 h, and ≥9 h per day, respectively) on the basis of their average daily sleep duration.

With respect to individual safety awareness, this section, including individual habitual safe behavior and consciousness, was composed of 15 questions (Cronbach’s α = 0.754). We scored totally (range: 3 to 36) and then classified the participants into three categories (low (i.e., ≤15), medium (i.e., 16–25), and high (i.e., ≥26)) according to their responses. Higher scores indicate a greater level of safety awareness. There were eight questions scored positively (i.e., (a) “Do you run and chase in the classroom?”; (b) “Do you run and chase on the way home or to school”; (c) “Do you run and chase in the schoolyard?”; (d) “Do you run and chase at home?”; (e) “Do you run and chase in the community?”; (f) “Do you have such behavior as climbing walls or trees?”; (g) “Do you play with classmates with stationery or sharp objects?”; (h) “Do you wear necklace or sharp objects when undertaking PA?”, and these eight questions share with the responses including “0 (would do often), 1 (would do seldom), and 2 (would never do)”, respectively), and the remaining seven were scored reversely (i.e., (a) “What do you do when you suffer from certain kind of disease that prohibits your participation in PA?”, including the responses “1 (tell the teachers truthfully), and 0 (conceal the truth)”; (b) “Will you look over the surroundings before undertaking PA?”, with the responses “3 (every time), 2 (often), 1 (sometimes), and 0 (never)”; (c) “What’s your opinion of warming up before undertaking PA?”, the responses include “3 (very important), 2 (important), 1 (unimportant), and 0 (very unimportant); (d) “What would you do if the teacher prohibits you from PA involvement out of safety concerns?”, with the responses ”1 (follow the teacher’s arrangement) and 0 (violate the teacher’s arrangement)”; (e) “Will you pay attention to your physical conditions like pulse or heartbeat during PA participation?”, include the responses “ 3 (every time), 2 (often), 1 (sometimes), and 0 (never)”; (f) “How do you treat physical examinations organized by school?”, the responses including “1 (take it seriously), and 0 (take it perfunctorily); (g) “Do you follow the rules and pay attention to others’ safety when undertaking PA?”, with the responses “3 (very often), 2 (often), 1 (sometimes) and 0 (never)”).

As to individual behavior when participating in PA, students were asked to complete 13 items (Cronbach’s α = 0.812). For example, “Would you do warming up before undertaking PA?”; “Would you exercise on wet/uneven floor, in insufficient lights, in the hottest/coldest hours, or with illness?”; “Would you wear suitable shoes/clothes, wear protective equipment, use sun proof, or drink water when undertaking PA?”; “Would you do cooling down after PA involvement?”. For each item, participants were required to indorse one of four responses: 1 (would never do), 2 (would hardly ever do), 3 (would do sometimes), 4 (would do often).

PARI, also known as sports and recreational activity-related injury, was defined as any injury resulting from participation in physical education (PE) class, sports activities or leisure time PA, such as sprain, fracture, sunstroke, and et al. [25]. In this study, a countable PARI event must occur during the past 12 months and meet at least one of the following four criteria: the student (1) has to immediately stop the PA and/or (2) cannot fully participate in the next planned PA and/or (3) is absent from class the next day and/or (4) needs to seek medical attention (from providers ranging from on-spot first aid personnel to general physicians or physiotherapists) [9,25]. All students were asked to report and count their PARI experiences according to the above four criteria. Pre-existing injuries, i.e., those occurred earlier than the past 12 months, were not counted as a PARI. In addition to ensuring that the self-reported injuries did meet these preceding criteria, we also captured the types of activity in which the injury occurred and the time of the injury occurrences. Furthermore, those injured students would be requested to provide information of PARI in each consequence category and other detailed PARI characteristics (including places, causes, injury types, injured body parts, and treatment, etc.). All of these were of help to further validate the outcome PARI measure.

### 2.3. Procedures 

This study was strictly conducted in accordance with the Declaration of Helsinki. Prior to approaching potential students, approval was obtained from the Shantou University Medical College ethics committee (SUMC-2016-22). Explanatory statement and parental consent forms were distributed to 3203 students, with a response rate being 97.6% (3127), and the questionnaires were subsequently given during school hours to the consenting students in the nominated classes. The purpose and meaning of our study was verbally explained to the students prior to their completion of the questionnaires, and our trained personnel would answer any questions of clarification as participants arose during the session. Nonetheless, 45 (1.44%) students were excluded owing to their serious incompletion of the questionnaires, resulting in a final valid sample size of 3082.

### 2.4. Statistical Analysis 

Descriptive statistics were performed to evaluate the characteristics of our participants. Continuous and categorical variables were presented as mean (standard deviation, SD) and number (percentage), and tested for between-group differences using independent-sample *t* tests and chi-square tests, respectively. The multivariate logistic regression model was used to consider all significant variables tested by *t* tests or chi-square tests together to estimate their risks for PARI, where odds ratios (ORs) and 95% confidence intervals (95% CIs) of variables kept in the final model were derived from. In the multivariate analyses, all variables were selected in forward manner (likelihood ratio, LR), with selection criteria of α_in_ = 0.05 and α_out_ = 0.10. SPSS 23.0 (SPSS, Inc., Chicago, IL, USA) was used for statistical analyses. A two-sided *p*-value less than 0.05 was considered statistically significant for statistical tests.

## 3. Results

Overall, the present study included 3082 eligible students (42.7% boys and 46.8% junior school students), with a mean age of 14.72 (SD: 1.54) years. About 775 students (25.1%) suffered from at least one PARI episode in the past 12 months, with significant differences being found in age, BMI, sports team member, sleep duration, individual safety awareness, father’s educational levels and parental marital status, and living with chronic conditions or not (all *p* < 0.05) between PARI and non-PARI. In addition, there were significantly different PARI occurrences in various study years and genders. Junior school students and boys experienced a higher proportion of PARI than their counterparts. Although less than 40% (38.0%) of participants were physically active according to the WHO recommendations on PA for youths [8], engagement in MVPA with different duration would significantly differ in injury experience (Table 1).

As presented in Table 2, the significant differences in characteristics of individual exercise behavior investigated between PARI and non-PARI included warming up before PA, regular drinking water during PA, wearing suitable shoes and protective equipment or with illness when involving in PA (all *p* < 0.05). Moreover, different activity environment would also influence the occurrence of PARI, with students being more prone to experience PARI while frequently exercising on wet/uneven floor, in insufficient lights, or in the hottest/coldest hours (all *p* < 0.05).

We finally performed a multivariate logistic regression analysis based on significant variables tested by *t* tests and chi-square tests together to estimate their odds ratios (ORs) for PARI, and the results of all significant variables kept in the final model were displayed in Table 3. Senior school students had smaller odds of sustaining PARI than junior ones, with girls being less likely to experience PARI compared with boys (OR = 0.598 and 0.744, respectively). Participation in PA with illness or on wet floor frequently would elevate the risk for injury, especially those with at least 120 min per day (adjusted ORs: 1.324–3.018). Students with stronger safety awareness were less vulnerable to suffer from injury during PA (OR = 0.311, 95% CI: 0.158~0.614). In addition, sports team membership, parental marital status, and living with any chronic condition or not were also significantly associated with PARI events.

Table 4 summarizes the numbers and consequences of PARI among 775 injured students stratified by gender. In the past 12 months, over one fifth of the injured students (21.3%, 165/775) experienced at least five PARI events (i.e., multiple injuries). Boys tended to easily suffer from multiple injuries than girls and were more likely to require medical care in accident and emergency department (A&E) or hospitalization, but less likely to have a time loss of class in the next day. Moreover, students in senior school had a significantly larger proportion of negative influence on the next PA participation (53.4% versus 35.3%, χ^2^ = 23.233, *p* < 0.001), while junior school students had a greater tendency to hospitalize due to PARI (8.5% versus 2.3%, χ^2^ = 12.012, *p* < 0.001) compared with their counterparts.

## 4. Discussion

A total of 3082 students (1316 boys and 1766 girls) from five middle schools in southern China participated in our survey. The overall incidence rate of PARI events in the past 12 months was 25.1%. Boys, junior school students, sports team member, those living with single parent, with lower safety awareness, or without chronic conditions were more likely to suffer from PARI. Less than 40% of participants were physically active. Nonetheless, participation in PA with longer duration (especially ≥120 min/d) would elevate the risk for injury, particularly those exercising on wet floor or with illness.

This study might be the first, to our knowledge, to present the problem of PARI among middle school students, showing that PARI is not uncommon among school-aged youths in southern China. We therefore should pay more attention to this serious issue in particular under the contemporary global promotion on a physically active lifestyle for health. PA has been proven to cause positive effects on individual fitness, but its inherent risks for injury should not be ignored. Apart from the financial medical burden, injuries could prejudice young people against ongoing participation in sports and recreational activities, resulting in the reduced PA level, and further be a barrier to promote an active lifestyle from childhood into adulthood [26,27]. Similar with other research [28], our results showed that the majority experienced a withdrawal time from PA due to the injuries, suggesting that effective injury-prevention measures should be implemented to reduce PARI among school-aged students who were recommended to participate in MVPA at least one hour per day.

Our study also revealed that boys were at higher risk for injury than girls, which was aligned with previous studies [29,30,31]. Several possibilities might be attributed to this gender discrepancy. Firstly, boys are more physically active than girls, which could be supported by our data (69.12 min/d versus 50.89 min/d). Secondly, owing to the differences in physical development at puberty (especially individual strength, speed and endurance) [32], boys prefer to involve in competitive team sports, such as basketball and football. The high rate of contact, sprinting and/or pivoting in these activities is the primary injury mechanism in sports [33,34]. Even in the same sport or recreational activity, boys are also more sensitive to sustain injuries because of their higher resistance and competitiveness with lower safety consciousness [35]. Thirdly, compared with girls, boys are easier to be impulsive with greater ambition, which elevates the potential risk for injury [34]. In addition, junior school students were more likely to suffer from injury than senior ones. The following reasons might contribute to this difference. First of all, adventure and impulsiveness inherent in younger students with relatively poor self-restraint might increase the risk for injury [32]. Moreover, poor individual safety awareness among junior students would also play a role. Data in our survey supported this explanation (22.06 versus 25.41 points on average). Collectively, boys and junior school students were more inclined to experience PARI events than their counterparts. There is an urgent call for practical prevention strategies to reduce PARI occurrence when promoting a physically active lifestyle.

The Chinese government has been making great efforts to promote youth sports to enhance their physical health in the past decades. Despite all these, our study showed that just nearly 38.0% of middle school students participated in MVPA at least 60 min per day. Researchers ascribed this low rate of PA involvement in part to the long homework time and electronic screen watching time among school-aged adolescents [36]. Being inactive is harmful to individual health, so is being too much active. Highly consistent with other studies [35,37,38], we found that PA participation with longer duration would significantly elevate the risks for injury, especially those who engaged in PA more than 120 min every day (OR = 3.018). Unfortunately, neither the WHO’s full reports on PA, nor other organization’s recommendations provide any suggestions on frequency and duration for safety on the basis of promotion and maintenance of physical health [6,7,10]. Moreover, sports team members in our study, who are involved in higher PA volume and level (78.63 min/d versus 55.69 min/d), were approximately 2-fold more likelihood to sustain PARI compared to their counterparts. Policy-makers and researchers should pay sufficient attention to the above results when globally promoting PA for the health of the public. Otherwise, the effectiveness of the global campaign and the benefits of PA would be compromised. Additionally, we also found that exercising on wet floor or with illness frequently would significantly increase the risk for injury, which was closely related with their poor individual safety awareness. While encouraging students to participate in PA actively, further theory-based multifaceted interventions aiming at reducing individual unsafe behaviors and providing safe environment for exercise are also urgent, in order to maximize the benefits of PA and reduce the risks of getting injured.

In this study, parental marital status and living with chronic conditions or not were also significantly associated with the occurrence of PARI events. More than 30% of students had eyesight impair (near-sight) among the whole sample, which limits them to involve in PA at higher frequency and level, and decreases the exposure risk for injury to some extent. This could be supported by our survey data (50.22 min/d for them and 60.71 min/d for their counterparts) and findings from other study [39]. Family context is of interest as potential injury determinant [12]. Students living in families whose parents were divorced or separated would potentially increase the possibility to sustain injuries. According to previous study, children raised by single parent have on average worse socio-psychological outcomes, physical health, and behavioral problems [40]. Moreover, lack of parental concerns might also contribute to the higher risk for injury experiences [41]. Further research could focus on these risk markers to identify the injury mechanism.

Several limitations of this study should be considered. First, the nature of cross-sectional study prevents us from making causal inference. Next, our data is self-reported, recall and report bias would be unavoidable. Except for the possibility of underreporting individual weight or over-reporting their height, for instance, students may remember major and later injuries clearly but forget minor and earlier ones easily. Although the measurement of the CLASS-C has sound reliability, PA, as a socially desirable individual behavior, is still likely to be over-reported. Our team group is conducting a prospective design to avoid the above limitations, whose results will be reported in the future. Finally, existence of missing data for the consequences of PARI would, to some extent, have a negative effect on the analysis of severity among injured students.

## 5. Conclusions

Despite the low participation rate of PA, PARI was prevalent among middle school students in southern China. We have identified several risk factors of PARI, including boys, junior school students, sports team member, those with higher PA levels, with lower safety awareness, living with single parent, without chronic conditions, and exercising on wet floor or with illness. Focused and effective actions should be taken therefore, with a full consideration of the above results presented in our study, to prevent middle school students from PARI and maximize the benefits of PA.

## Figures and Tables

**Table 1:** Comparison of socio-demographic characteristics and other main factors in participants with physical activity-related injury (PARI) or not.

Characteristics	All (*N* = 3082) *n* (%)	Non-PARI (*N* = 2307) *n* (%)	PARI (*N* = 775) *n* (%)	χ^2^/t *	*p*-Value
Study year				24.425	<0.001
Junior	1442 (46.8)	1020 (70.7)	422 (29.3)		
Senior	1640 (53.2)	1287 (78.5)	353 (21.5)		
Gender				28.033	<0.001
Boy	1316 (42.7)	922 (70.1)	394 (29.9)		
Girl	1766 (57.3)	1385 (78.4)	381 (21.6)		
Sports team member				5.764	0.016
No	2788 (97.0)	2081 (74.6)	707 (25.4)		
Yes	87 (3.0)	55 (63.2)	32 (36.8)		
Participation in MVPA ^1^				84.960	<0.001
<60 min/d	1910 (62.0)	1523 (79.8)	387 (20.2)		
60–<120 min/d	803 (26.1)	569 (70.9)	234 (29.1)		
≥120 min/d	369 (12.0)	215 (58.3)	154 (41.7)		
Sleep duration				13.486	0.009
<6 h/d	153 (5.9)	103 (67.3)	50 (32.7)		
6–<7 h/d	378 (14.5)	289 (76.5)	89 (23.5)		
7–<8 h/d	748 (28.7)	564 (75.4)	184 (24.6)		
8–<9 h/d	803 (30.8)	607 (75.6)	196 (24.4)		
≥9.00 h/d	528 (20.2)	364 (68.9)	164 (31.1)		
Chronic disease/symptom				14.629	<0.001
No	1736 (59.7)	1248 (71.9)	488 (28.1)		
Yes	1174 (40.3)	918 (78.2)	256 (21.8)		
Safety awareness (points)				22.060	<0.001
Low (≤15)	94 (3.1)	52 (55.3)	42 (44.7)		
Medium (16–25)	1677 (55.9)	1263 (75.3)	414 (24.7)		
High (≥26)	1229 (41.0)	946 (76.9)	283 (23.1)		
Father’s educational levels				7.608	0.022
Primary school or below	853 (27.7)	609 (71.4)	244 (28.6)		
Middle school Vocational school or above	1953 (63.4) 276 (9.0)	1490 (76.3) 208 (75.4)	463 (23.7) 68 (24.6)		
Mother’s educational levels				1.159	0.560
Primary school or below	1420 (46.1)	1050 (73.9)	370 (26.1)		
Middle school Vocational school or above	1453 (47.1) 209 (6.8)	1099 (75.6) 158 (75.6)	354 (24.4) 51 (24.4)		
Parental marital status				8.277	0.004
Married	2696 (87.5)	2041 (75.7)	655 (24.3)		
Others ^2^	386 (12.5)	266 (68.9)	120 (31.1)		
Age ($\bar{x}$ ± s, years)	14.72 ± 1.54	14.80 ± 1.54	14.48 ± 1.51	4.968	<0.001
BMI ^3^ ($\bar{x}$ ± s, kg/m^2^)	18.47 ± 2.72	18.58 ± 2.71	18.16 ± 2.70	3.440	0.001

* Categorical variables (all variables except for age and BMI) were tested by chi-square tests, while continuous variables (age and BMI) were tested by independent-sample *t* tests; ^1^: MVPA, moderate- to vigorous-intensity physical activity; ^2^: including divorce, separation or widow, et al; ^3^: BMI, body mass index.

**Table 2:** Comparison of individual behavioral factors investigated involvement in physical activity (PA) in participants with physical activity-related injury (PARI) or not.

Characteristics	All (*N* = 3082) *n* (%)	Non-PARI (*N* = 2307) *n* (%)	PARI (*N* = 775) *n* (%)	χ^2^	*p*-Value
Warming up				12.311	0.006
Never	742 (24.1)	571 (77.0)	171 (23.0)		
Seldom	1325 (43.0)	980 (74.0)	345 (26.0)		
Sometimes	636 (20.6)	492 (77.4)	144 (22.6)		
Often	379 (12.3)	264 (69.7)	115 (30.3)		
Cooling down				4.058	0.255
Never	1096 (35.6)	843 (76.9)	253 (23.1)		
Seldom	1330 (43.2)	985 (74.1)	345 (25.9)		
Sometimes	450 (14.6)	327 (72.7)	123 (27.3)		
Often	206 (6.7)	152 (73.8)	54 (26.2)		
Sun proof				0.325	0.955
Never	1973 (64.0)	1465 (74.3)	508 (25.7)		
Seldom	658 (21.3)	497 (75.5)	161 (24.5)		
Sometimes	287 (9.3)	220 (76.7)	67 (23.3)		
Often	164 (5.3)	125 (76.2)	39 (23.8)		
Regular drinking				9.387	0.025
Never	414 (13.4)	333 (80.4)	81 (19.6)		
Seldom	734 (23.8)	559 (76.2)	175 (23.8)		
Sometimes	1087 (35.3)	799 (73.5)	288 (26.5)		
Often	847 (27.5)	616 (72.7)	231 (27.3)		
Suitable shoes				8.456	0.037
Never	605 (19.6)	477 (78.8)	128 (21.2)		
Seldom	683 (22.2)	507 (74.2)	176 (25.8)		
Sometimes	758 (24.6)	567 (74.8)	191 (25.2)		
Often	1036 (33.6)	756 (73.0)	280 (27.0)		
Suitable clothes				4.962	0.175
Never	930 (30.2)	717 (77.1)	213 (22.9)		
Seldom	1000 (32.4)	739 (73.9)	261 (26.1)		
Sometimes	576 (18.7)	428 (74.3)	148 (25.7)		
Often	576 (18.7)	423 (73.4)	153 (26.6)		
Protective equipment				8.637	0.013
Never	2447 (79.4)	1861 (76.1)	586 (23.9)		
Seldom	483 (15.7)	343 (71.0)	140 (29.0)		
Sometimes/Often	152 (4.9)	103 (67.8)	49 (32.2)		
Wet floor				27.982	<0.001
Never	2091 (67.8)	1602 (76.6)	489 (23.4)		
Seldom	795 (25.8)	577 (72.6)	218 (27.4)		
Sometimes/Often	196 (6.4)	128 (65.3)	68 (34.7)		
Uneven floor				28.371	<0.001
Never	1832 (59.4)	1426 (77.8)	406 (22.2)		
Seldom	921 (29.9)	644 (69.9)	277 (30.1)		
Sometimes	242 (7.9)	172 (71.1)	187 (28.9)		
Often	87 (2.8)	65 (74.7)	22 (25.3)		
Insufficient lights				13.200	0.004
Never	1096 (35.6)	840 (76.6)	256 (23.4)		
Seldom	1385 (44.9)	1032 (74.5)	353 (25.5)		
Sometimes	477 (15.5)	355 (74.4)	122 (25.6)		
Often	124 (4.0)	80 (64.5)	44 (35.5)		
Hottest hours				25.535	<0.001
Never	1384 (44.9)	1083 (78.3)	301 (21.7)		
Seldom	1259 (40.9)	937 (74.4)	322 (25.6)		
Sometimes	349 (11.3)	226 (64.8)	123 (35.2)		
Often	90 (2.9)	61 (67.8)	29 (32.2)		
Coldest hours				22.218	<0.001
Never	850 (27.6)	683 (80.4)	167 (19.6)		
Seldom	1350 (43.8)	1015 (75.2)	335 (24.8)		
Sometimes	679 (22.0)	476 (70.1)	203 (29.9)		
Often	203 (6.6)	133 (65.5)	70 (34.5)		
Illness				21.449	<0.001
Never	2311 (75.0)	1766 (76.4)	545 (23.6)		
Seldom	585 (19.0)	408 (69.7)	177 (30.3)		
Sometimes/Often	186 (6.0)	133 (71.5)	53 (28.5)		

**Table 3:** Multivariate logistic regression to estimate risks of potential factors investigated for physical activity-related injury (PARI) among middle school students in Southern China.

Characteristics	*β*	*Wald*	*p*-Value	Adjusted OR	95% CI
Gender					
Boy				1 (ref.)	
Girl	−0.296	4.872	0.027	0.744	0.572~0.967
Study year					
Junior				1 (ref.)	
Senior	−0.455	9.703	<0.001	0.598	0.453~0.790
Sports team member					
No				1 (ref.)	
Yes	0.702	4.463	0.035	2.017	1.052~3.868
Participation in MVPA ^1^					
<60 min/d				1 (ref.)	
60–<120 min/d	0.508	11.572	<0.001	1.668	1.255~2.215
≥120 min/d	1.107	37.180	<0.001	3.018	2.119~4.299
Safety awareness (points)					
Low (≤15)				1 (ref.)	
Medium (16–25)	−0.972	5.850	0.001	0.370	0.185~0.743
High (≥26)	−1.167	8.793	0.005	0.311	0.158~0.614
Chronic disease/symptom					
No				1 (ref.)	
Yes	−0.405	8.974	0.002	0.663	0.511~0.861
Parental marital status					
Married				1 (ref.)	
Others ^2^	0.590	6.320	0.009	1.791	1.155~2.777
Wet floor					
Never				1 (ref.)	
Seldom	0.360	3.632	0.003	1.324	1.110~1.595
Sometimes	0.373	4.940	<0.001	1.944	1.378~2.742
Often	0.622	5.811	0.038	1.940	1.036~3.632
Illness					
Never				1 (ref.)	
Seldom	0.460	9.839	0.002	1.576	1.182~2.103
Sometimes/Often	0.549	6.198	0.241	1.733	0.886~1.850

^1^: MVPA, moderate- to vigorous-intensity physical activity; ^2^: including divorce, separation or widow, et al.

**Table 4:** Numbers and consequences of physical activity-related injury (PARI) between boys and girls.

Characteristics	All (*N* = 775) *n* (%)	Gender		χ^2^	*p*-Value
		Boys (*N* = 394) *n* (%)	Girls (*N* = 381) *n* (%)		
Number of PARI episodes				16.463	<0.001
1–4	610 (78.7)	287 (72.8)	323 (84.8)		
≥5	165 (21.3)	107 (27.2)	58 (15.2)		
Consequences of PARI ^a^					
Immediately stop the PA				0.265	0.607
No	262 (34.0)	130 (33.2)	132 (34.9)		
Yes	508 (66.0)	262 (66.8)	246 (65.1)		
Absent from the next PA				0.674	0.412
No	399 (57.7)	210 (58.3)	189 (55.3)		
Yes	293 (42.3)	140 (41.7)	153 (44.7)		
Class absence next day				5.824	0.016
No	522 (74.5)	282 (78.3)	240 (70.4)		
Yes	179 (25.5)	78 (21.7)	101 (29.6)		
Require first aid				0.228	0.633
No	634 (90.3)	327 (90.8)	307 (89.8)		
Yes	68 (9.7)	33 (9.2)	35 (10.2)		
Treatment in A&E ^1^				9.258	0.002
No	621 (88.6)	307 (85.0)	314 (92.4)		
Yes	80 (11.4)	54 (15.0)	26 (7.6)		
Treatment in non-A&E				0.025	0.875
No	567 (80.9)	292 (81.1)	275 (80.6)		
Yes	134 (19.1)	68 (18.9)	66 (19.4)		
Overnight in hospital				3.129	0.077
No	662 (94.3)	335 (92.8)	327 (95.9)		
Yes	40 (5.7)	26 (7.2)	14 (4.1)		
Hospitalization				6.450	0.011
No	660 (94.2)	332 (92.0)	328 (96.5)		
Yes	41 (5.8)	29 (8.0)	12 (3.5)		

^1^: A&E, accident and emergency department; ^a^: The sum of the categories might not equal the total number of PARI episodes because of small amounts of missing data for some variables.

## References

[1] Hallal P.C., Victora C.G., Azevedo M.R., Wells J.C. (2006). Adolescent physical activity and health: A systematic review. Sports Med..

[2] Hallal P.C., Dumith S.C., Reichert F.F., Menezes A.M., Araújo C.L., Wells J.C., Ekelund U., Victora C.G. (2011). Cross-sectional and longitudinal associations between physical activity and blood pressure in adolescence: Birth cohort study. J. Phys. Act. Health.

[3] Nevill A.M., Duncan M.J., Lahart I.M., Sandercock G. (2017). Cardiorespiratory fitness and activity explains the obesity-deprivation relationship in children. Health Promot. Int..

[4] Penedo F.J., Dahn J.R. (2005). Exercise and well-being: A review of mental and physical health benefits associated with physical activity. Curr. Opin. Psychiatry.

[5] Education C.N. (2007). Suggestion of CPC Central Committee and State Council on strengthing the youth sports to enhance youth physical. Chin. J. Sch. Health.

[6] Haskell W.L., Lee I.M., Pate R.R., Powell K.E., Blair S.N., Franklin B.A., Macera C.A., Heath G.W., Thompson P.D., Bauman A. (2007). Physical activity and public health: Updated recommendation for adults from the American College of Sports Medicine and the American Heart Association. Med. Sci. Sports Exerc..

[7] Office of Disease Prevention and Health Promotion Physical Activity Guildelines for Children and Adolescents. https://health.gov/paguidelines/guidelines/children.aspx.

[8] World Health Organization Physical Activity and Young People. Recommended Levels for Physical Activity for Children Aged 5–17 Years. www.who.int/dietphysicalactivity/factsheet_young_people/en/.

[9] Bloemers F., Collard D., Paw M.C.A., Van Mechelen W., Twisk J., Verhagen E. (2012). Physical inactivity is a risk factor for physical activity-related injuries in children. Br. J. Sports Med..

[10] World Health Organization Global Recommendations on Physical Activity for Health. http://apps.who.int/iris/bitstream/10665/44399/1/9789241599979_eng.pdf.

[11] King M.A., Pickett W., King A.J. (1998). Injury in Canadian youth: A secondary analysis of the 1993–1994 Health Behaviour in School-Aged Children Survey. Can. J. Public Health.

[12] Pickett W., Molcho M., Simpson K., Janssen I., Kuntsche E., Mazur J., Harel Y., Boyce W.F. (2005). Cross national study of injury and social determinants in adolescents. Inj. Prev..

[13] Brudvik C., Hove L.M. (2003). Childhood fractures in Bergen, Norway: Identifying high-risk groups and activities. J. Pediatr. Orthop..

[14] Hedström E.M., Svensson O., Bergström U., Michno P. (2010). Epidemiology of fractures in children and adolescents. Acta Orthop..

[15] Collard D.C., Verhagen E.A., Mechelen W.V., Heymans M.W., Chinapaw M.J. (2011). Economic burden of physical activity-related injuries in Dutch children aged 10–12. Br. J. Sports Med..

[16] Finch C., Cassell E. (2006). The public health impact of injury during sport and active recreation. J. Sci. Med. Sport.

[17] Finch C., Owen N., Price R. (2001). Current injury or disability as a barrier to being more physically active. Med. Sci. Sports Exerc.

[18] Grimmer K.A., Jones D., Williams J. (2000). Prevalence of adolescent injury from recreational exercise: An Australian perspective. J. Adolesc. Health.

[19] Trost S.G., Sallis J.F., Pate R.R., Freedson P.S., Taylor W.C., Dowda M. (2003). Evaluating a model of parental influence on youth physical activity. Am. J. Prev. Med..

[20] Flynn J.M., Lou J.E., Ganley T.J. (2002). Prevention of sports injuries in children. Curr. Opin. Pediatr..

[21] Siesmaa E., Blitvich J., Telford A., Finch C., Farelli A. (2011). Factors that are most influential in children’s continued and discontinued participation in organised sport: The role of injury and injury risk perceptions. Sport Participation: Health Benefits, Injuries, and Psychological Effects.

[22] Yin M.M., Wang S.M., Zhuang J., Chen P.J., Zou J.L., Yuan D.G. (2011). Analysis on sports injuries of adolescents in Shanghai. Chin. J. Sch. Health.

[23] Li Q.L., Zou L.C., Zhang S.S., Guan S. (2015). Epidemiological Study on Sports Injuries among Adolescents in Guangzhou. J. Guangzhou Sport Univ..

[24] Li H.Y., Chen P.J., Zhuang J. (2011). Revision and reliability validity assessment of Children’s Leisure Activities Study Survey. Chin. J. Sch. Health.

[25] Gao Y., Lo Y.M., Duan Y.P., Lee K.L. (2015). A pilot study on physical activity related injury (PARI) in primary school children in Hong Kong. Inj. Med. Inernet.

[26] Gignac M.A., Cao X., Ramanathan S., White L.M., Hurtig M., Kunz M., Marks P.H. (2015). Perceived personal importance of exercise and fears of re-injury: A longitudinal study of psychological factors related to activity after anterior cruciate ligament reconstruction. BMC Sports Sci. Med. Rehabil..

[27] Emery C., Tyreman H. (2009). Sport participation, sport injury, risk factors and sport safety practices in Calgary and area junior high schools. Paediatr. Child Health.

[28] Hansen A.R., Pritchard T., Melnic I., Zhang J. (2016). Physical activity, screen-time, and school absenteeism: Self-reports from NHANES 2005–2008. Curr. Med. Res. Opin..

[29] Gutierrez G., Sills M., Bublitz C.D., Westfall J.M. (2006). Sports-related injuries in the United States: Who gets care and who does not. Clin. J. Sport Med..

[30] Deits J., Yard E.E., Collins C.L., Fields S.K., Comstock R.D. (2010). Patients with Ice Hockey Injuries Presenting to US Emergency Departments, 1990–2006. J. Athl. Train..

[31] Gilchrist J., Haileyesus T., Murphy M.W., Yard E.E. (2011). Nonfatal Sports and Recreation Heat Illness Treated in Hospital Emergency Departments—United States, 2001–2009. MMWR Morb. Mortal. Wkly. Rep..

[32] Spinks A.B., Mcclure R.J. (2007). Quantifying the risk of sports injury: A systematic review of activity-specific rates for children under 16 years of age. Br. J. Sports Med..

[33] Emery C.A. (2003). Risk factors for injury in child and adolescent sport: A systematic review of the literature. Clin. J. Sport Med..

[34] Peden M., Oyegbite K., Ozannesmith J., Hyder A.A., Branche C., Rahman A.K.M.F., Rivara F., Bartolomeos K., Peden M., Oyegbite K. (2008). World report on child injury prevention. Inj. Prev..

[35] Pons-Villanueva J., Segui-Gomez M., Martinez-Gonzalez M.A. (2010). Risk of injury according to participation in specific physical activities: A 6-year follow-up of 14 356 participants of the SUN cohort. Int. J. Epidemiol..

[36] Wang Z.H., Dong Y.H., Song Y., Yang Z.P., Ma J. (2017). Analysis on prevalence of physical activity time <1 hour and related factors in students aged 9–22 years in China, 2014. Chin. J. Epidemiol..

[37] Parkkari J., Kannus P., Natri A., Lapinleimu I., Palvanen M., Heiskanen M., Vuori I., Järvinen M. (2004). Active living and injury risk. Int. J. Sports Med..

[38] Jones B.H., Cowan D.N., Tomlinson J.P., Robinson J.R., Polly D.W., Frykman P.N. (1993). Epidemiology of injuries associated with physical training among young men in the army. Med. Sci. Sports Exerc..

[39] Ahn S.H., Um Y.J., Kim Y.J., Kim H.J., Oh S.W., Lee C.M., Kwon H., Joh H.K. (2016). Association between physical activity levels and physical symptoms or illness among university students in Korea. Korean J. Fam. Med..

[40] Scharte M., Bolte G., GME Study Group (2013). Increased health risks of children with single mothers: The impact of socio-economic and environmental factors. Eur. J. Public Health.

[41] Telford A., Finch C.F., Barnett L., Abbott G., Salmon J. (2012). Do parents’ and children’s concerns about sports safety and injury risk relate to how much physical activity children do?. Br. J. Sports Med..

